# Supplementary material for: High-temperature stability in air of Ti3C2Tx MXene-based composite with extracted bentonite
Source: Nat Commun. 2022 Sep 22;13:5551. doi: 10.1038/s41467-022-33280-2 (PMC9499972; doi:10.1038/s41467-022-33280-2)
Supplement: Supplementary file 1 — Supplementary Information [file 41467_2022_33280_MOESM1_ESM.pdf]

Supplementary Information

High-temperature stability in air of $Ti_3C_2T_x$ MXene-based composite with extracted bentonite

Na Liu,[1, 2, 3] Qiaoqiao Li,[4] Hujie Wan,[1, 2] Libo Chang,[1, 2] Hao Wang,[5] Jianhua Fang,[3] Tianpeng Ding,[1, 2] Qiye Wen,[1, 2,*] Liujiang Zhou,[2, 4,*] Xu Xiao[1, 2,*]

[1] School of Electronic Science and Engineering, State Key Laboratory of Electronic Thin Film and Integrated Devices, University of Electronic Science and Technology of China, Chengdu, Sichuan 610054, China.

[2] Yangtze Delta Region Institute (Huzhou), University of Electronic Science and Technology of China, Huzhou, Zhejiang 313001, China.

[3] Department of Petroleum, Oil and Lubricants, Army Logistic Academy of PLA, Chongqing 401331, China.

[4] School of Physics, University of Electronic Science and Technology of China, Chengdu, Sichuan 610054, China.

[5] Research Institute of Superconductor Electronics, School of Electronic Science and Engineering, Nanjing University, Nanjing 210023, China.

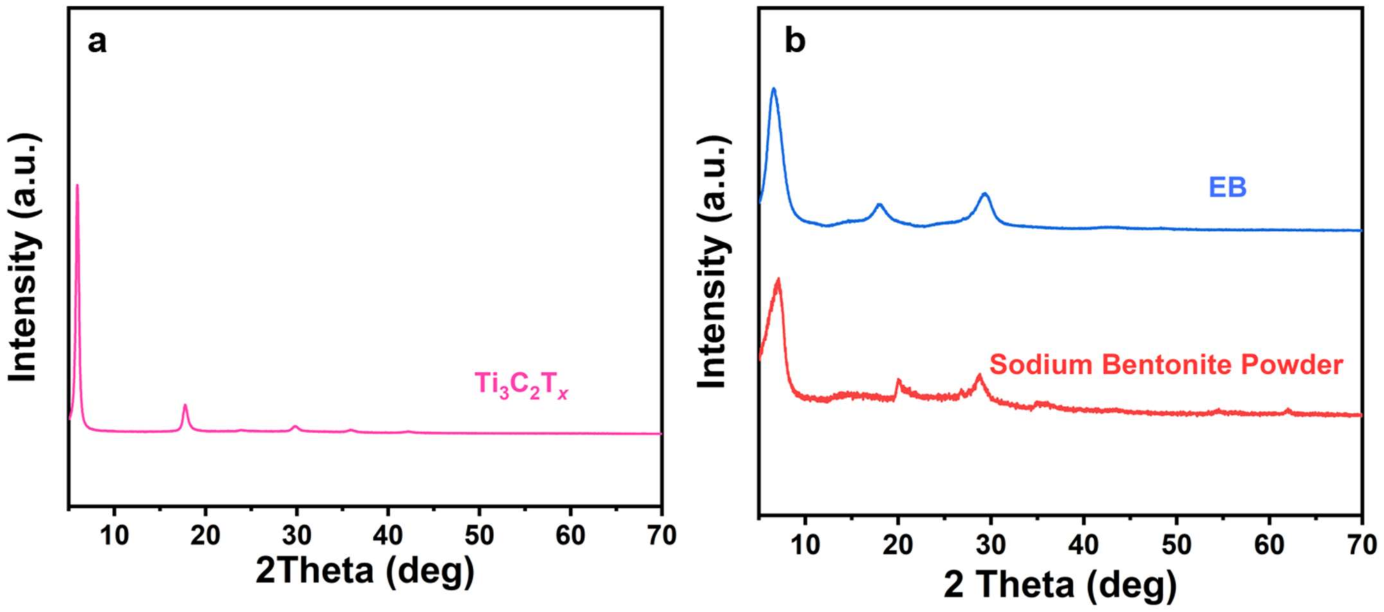

Fig. S1 XRD patterns of $Ti_3C_2T_x$ (a), Sodium Bentonite powder and EB (b). Source data are provided as a Source Data file.

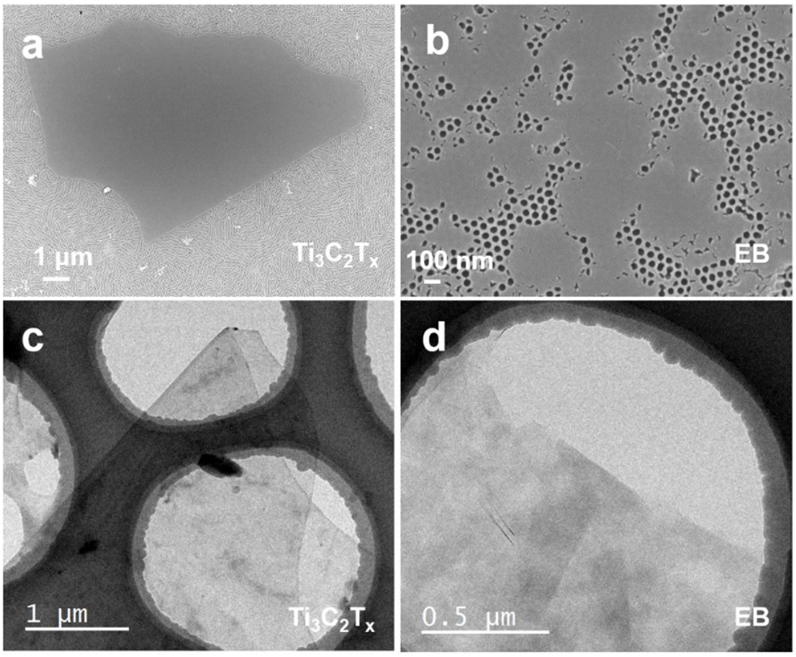

Fig. S2 SEM images of $Ti_3C_2T_x$ plate (a) and EB plate (b); TEM images of $Ti_3C_2T_x$ plate (c) and EB plate (d).

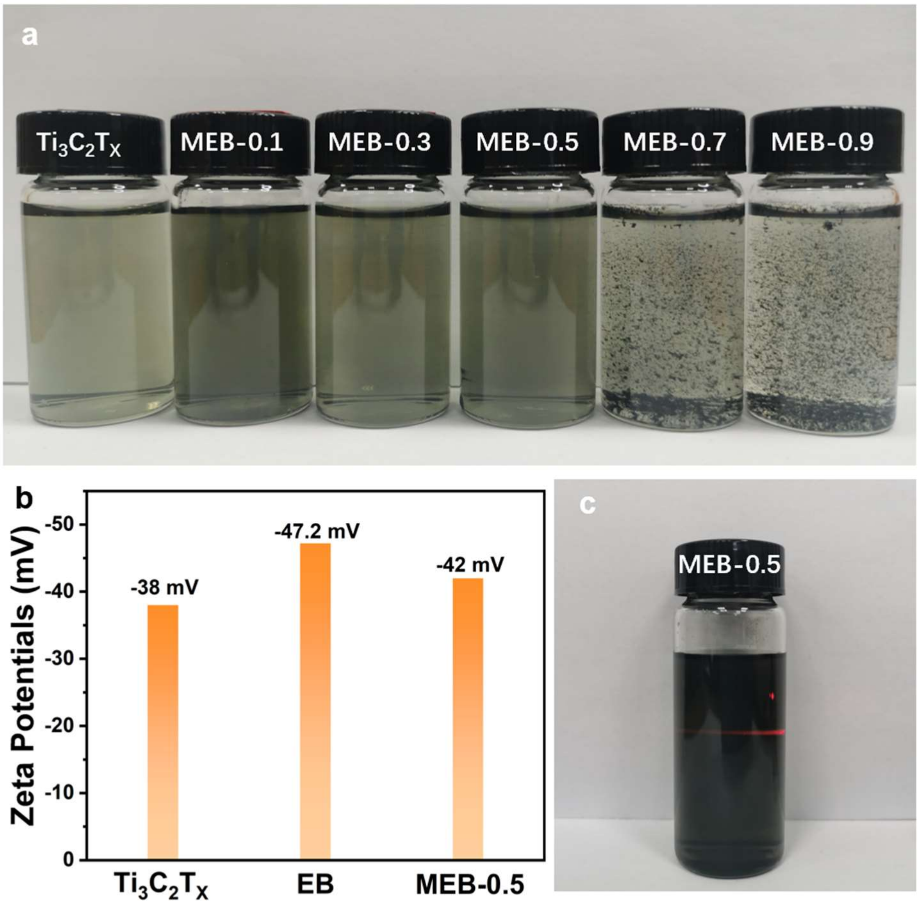

Fig. S3 (a) Photograph of pristine $Ti_3C_2T_x$ and the MEB aqueous dispersions (with 10, 30, 50, 70, 90 wt.% EB which are the dispersions to prepare MEB-0.1, MEB-0.3, MEB-0.5, MEB-0.7, MEB-0.9, respectively). (b) Zeta potentials of $Ti_3C_2T_x$, EB and MEB-0.5 aqueous dispersions. (c) The Tyndall effect of MEB-0.5 aqueous dispersion. Source data are provided as a Source Data file.

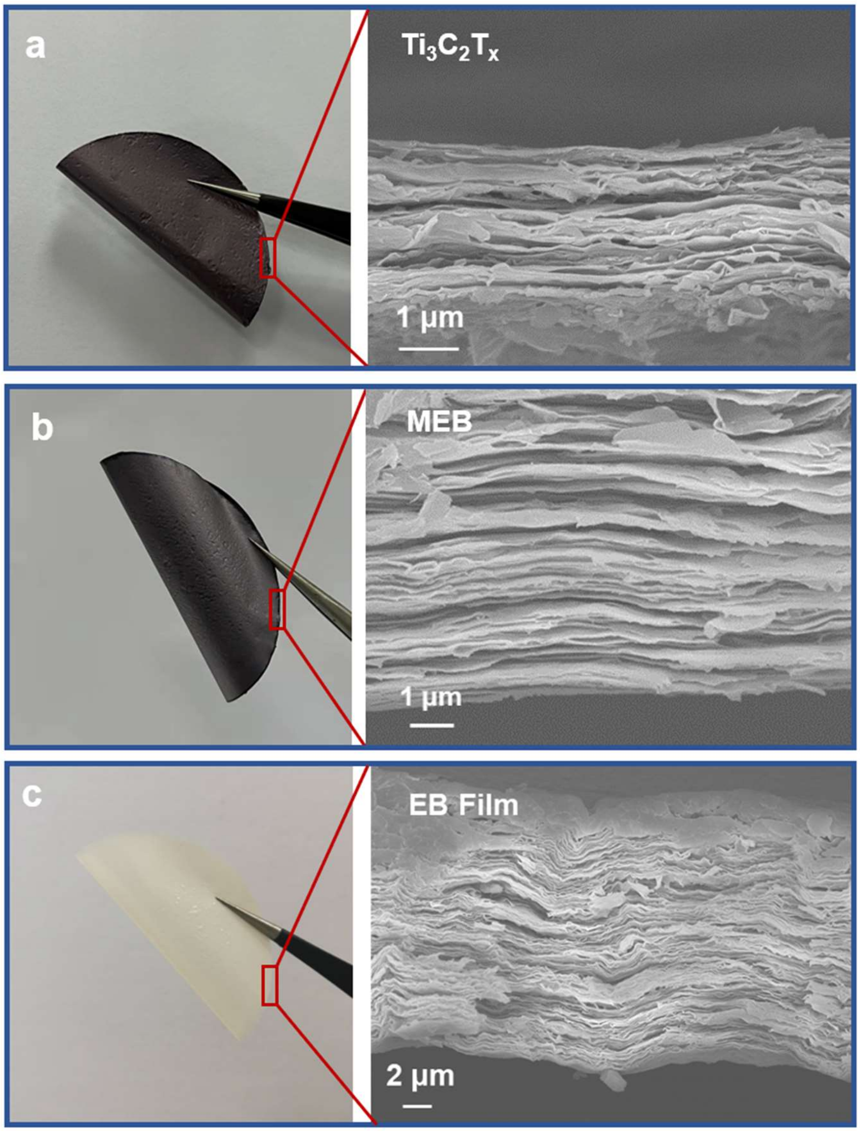

Fig. S4 Photographs of $Ti_3C_2T_x$ film (a), MEB film (b), EB film(c), exhibiting the flexibility and the corresponding cross-sectional SEM images

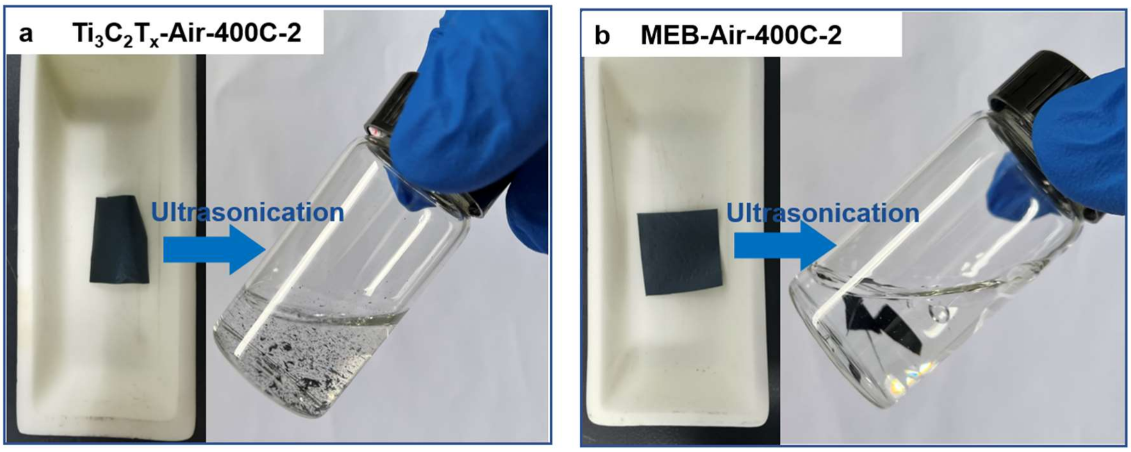

Fig. S5 Photographs of the films annealed in synthetic air at 400 °C for 2 hours, and be treated by ultrasonication for 1min in water: (a) $Ti_3C_2T_x$-Air-400C-2 (b) MEB-Air-400C-2.

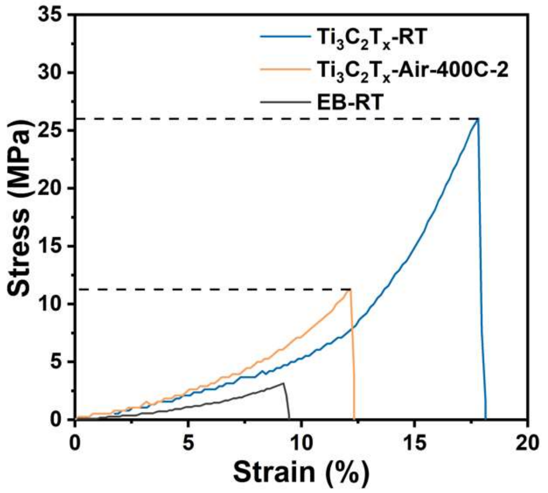

Fig. S6 The tensile stress-strain curves of $Ti_3C_2T_x$-RT, $Ti_3C_2T_x$-Air-400C-2, and EB-RT. Source data are provided as a Source Data file.

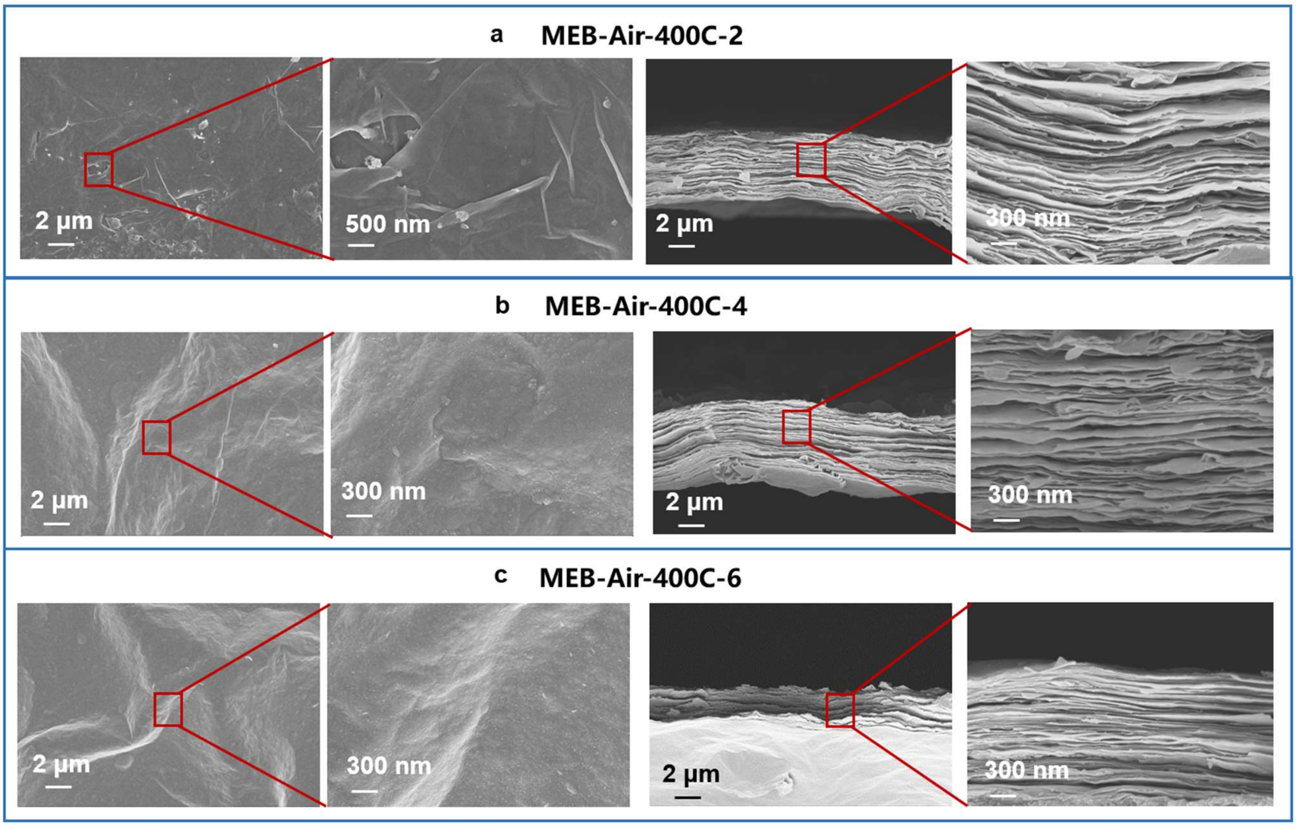

Fig. S7 SEM images of the surface and cross-section of different samples: (a) MEB-Air-400C-2; (b) MEB-Air-400C-4; (c) MEB-Air-400C-6.

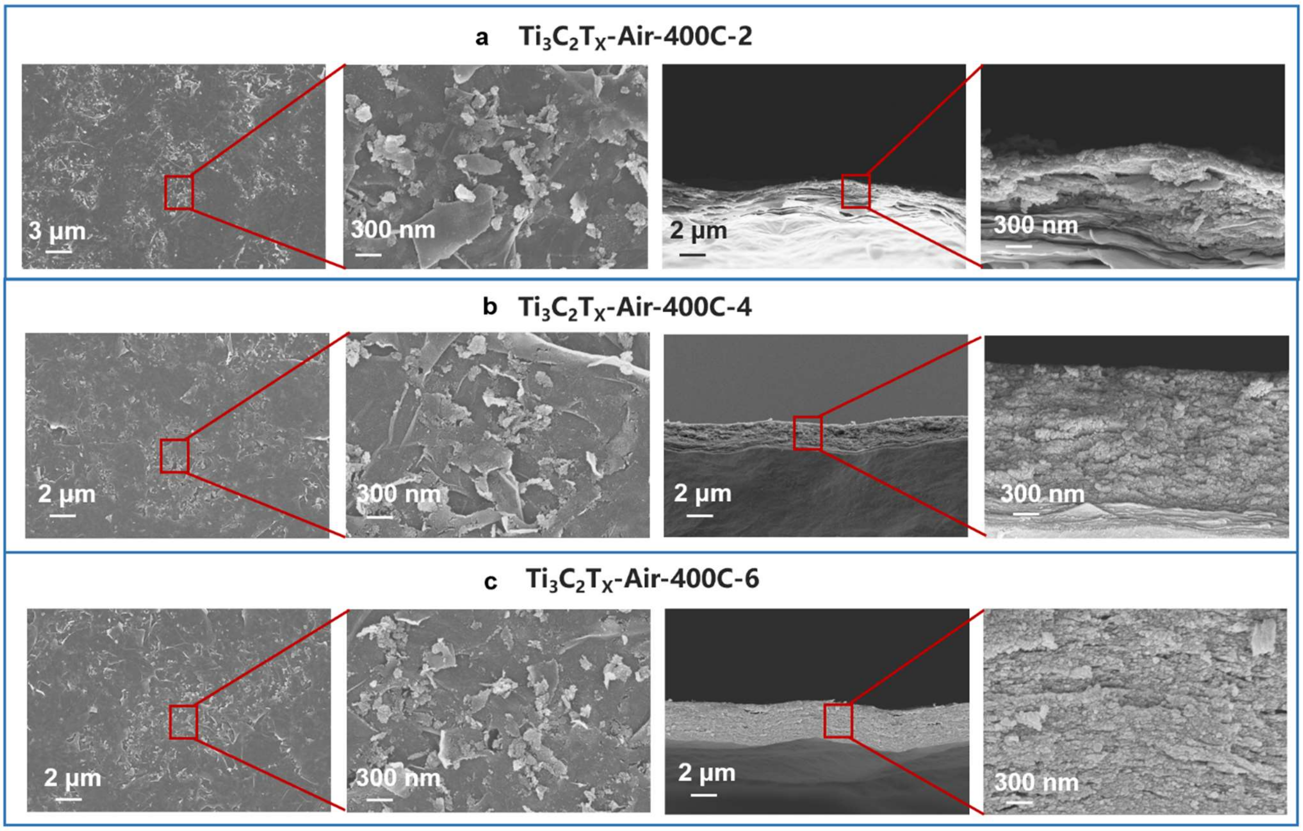

Fig. S8 SEM images of the surface and cross-section of different samples: (a) $Ti_3C_2T_x$-Air-400C-2; (b) $Ti_3C_2T_x$-Air-400C-4; (c) $Ti_3C_2T_x$-Air-400C-6.

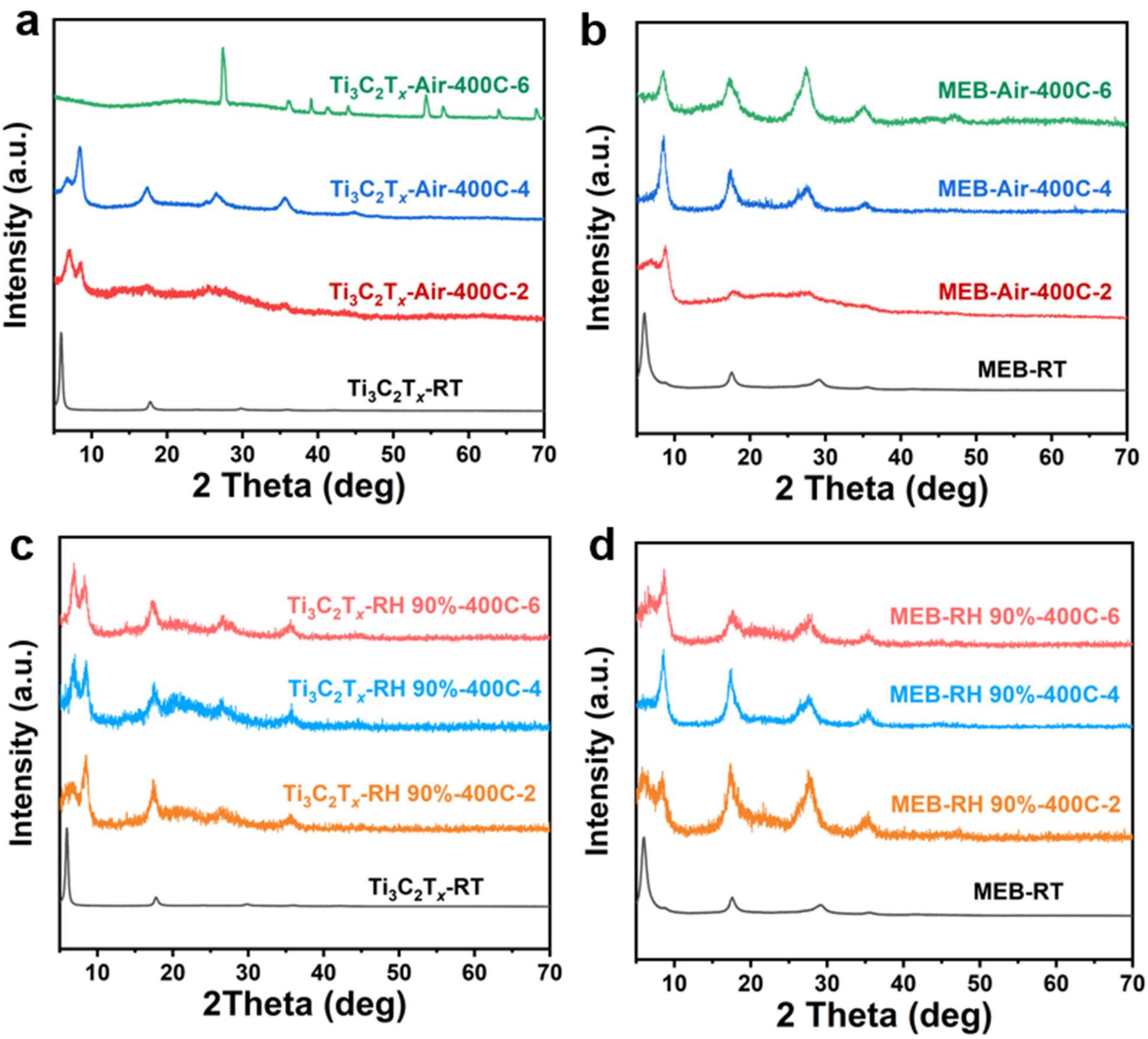

Fig. S9 XRD patterns of $Ti_3C_2T_x$ and MEB before and after annealing under different conditions. Source data are provided as a Source Data file.

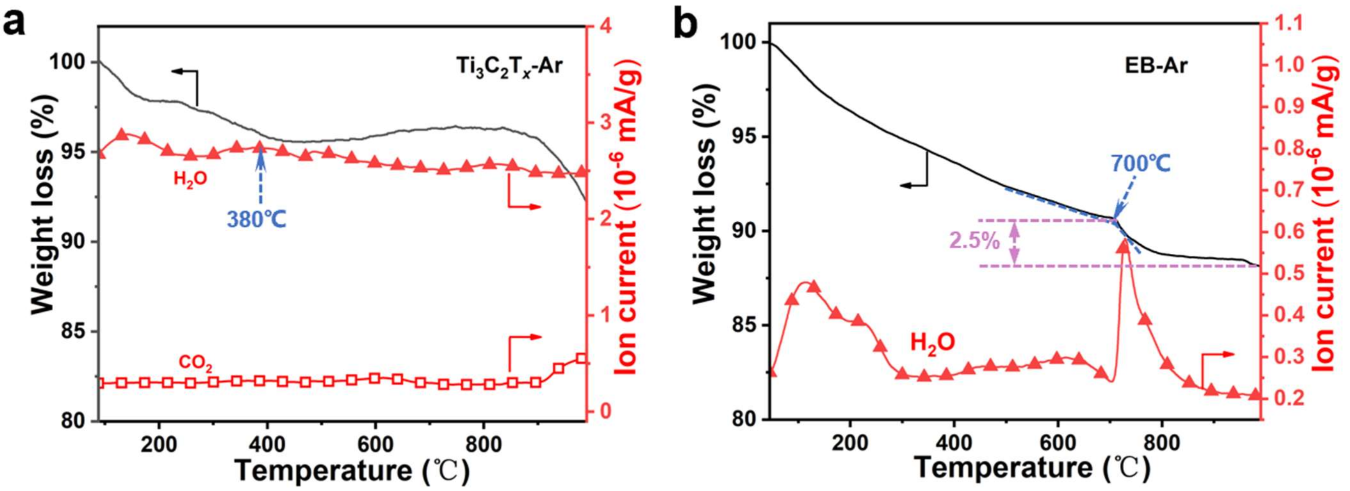

Fig. S10 TG curves in Ar with mass spectrometry analysis (MS) for the atomic mass unit (amu) of 18/H_2O and 44/CO_2. (a) $Ti_3C_2T_x$ (b) EB. Source data are provided as a Source Data file.

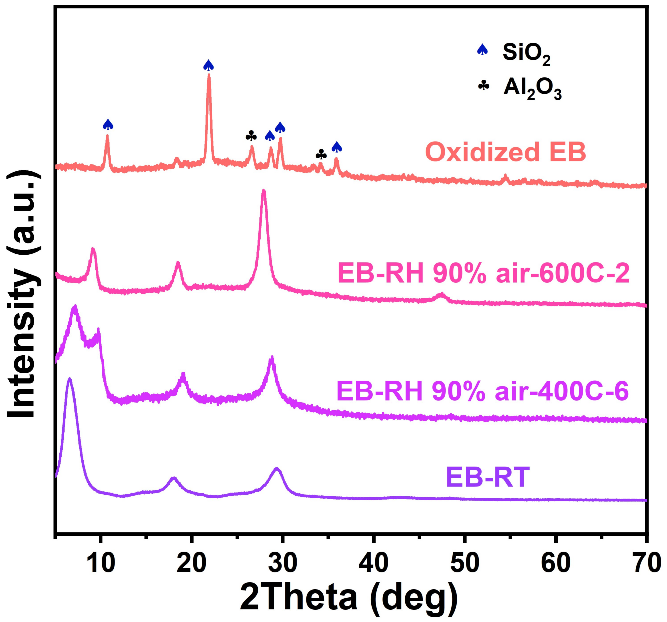

Fig. S11 XRD patterns of oxidized EB and EB after annealing under different conditions. Source data are provided as a Source Data file.

XRD patterns display the reduction of the interlayer (*d*-) spacing, characterized by the peak of (002) shifting from 2θ = 6.0° to ~ 8.0° after annealing at 600°C for 2 h. SiO_2 and Al_2O_3 characteristic peaks are not observed after treatments, which further suggests the thermal stability of EB in our annealing experiments with the presence of O_2 and H_2O.

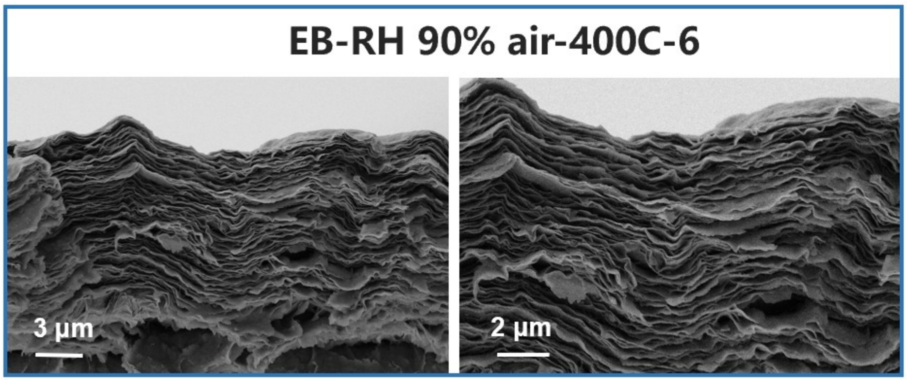

Fig. S12 SEM images of the cross-section of EB-RH 90% air-400C-6. Layered structure of EB retains after annealing at 400℃ for 6 h with the presence of H_2O and O_2.

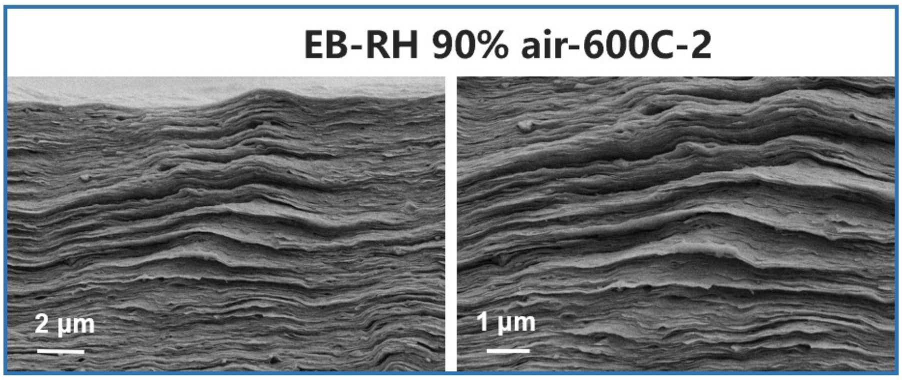

Fig. S13 SEM images of the cross-section of EB-RH 90% air-600C-2. Layered structure of EB retains after annealing at 600℃ for 2 h with the presence of H_2O and O_2.

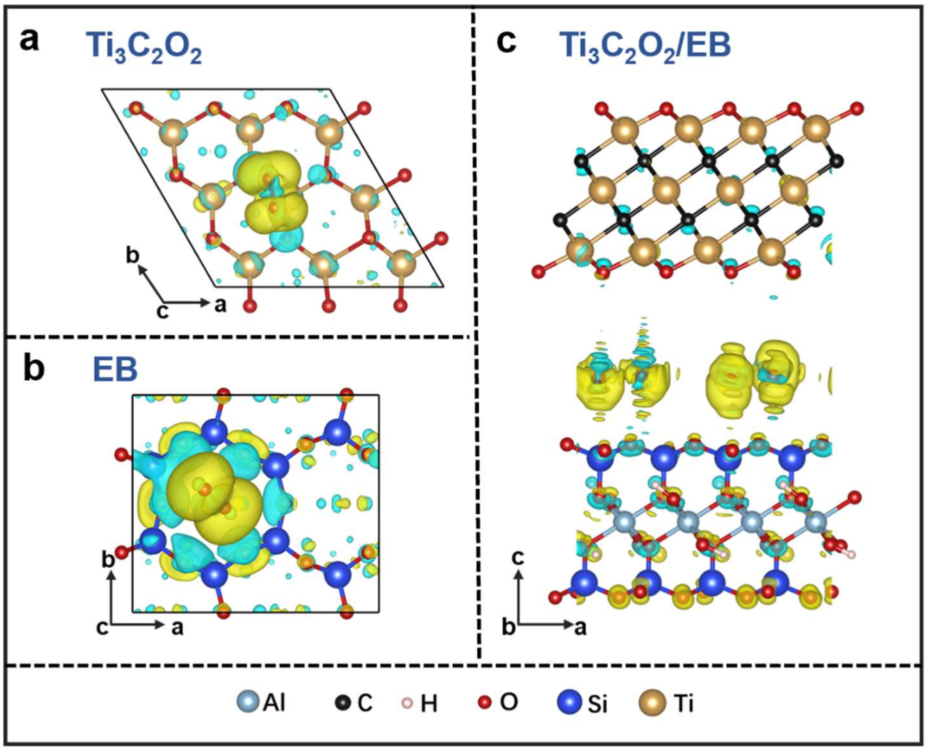

Fig. S14 The top views of charge density difference plots for the stable configurations of O_2 adsorbed on (a) $Ti_3C_2O_2$ and (b) EB. (c) The charge density plot for saturated adsorption of O_2 on EB in $Ti_3C_2O_2$/EB heterojunction. The isosurface level is set to be 0.0002 e/Å^3 except that for O_2 adsorbed on EB with a value of 0.0006 e/Å^3. The yellow area indicates the charge accumulation, and the green region represents charge depletion.

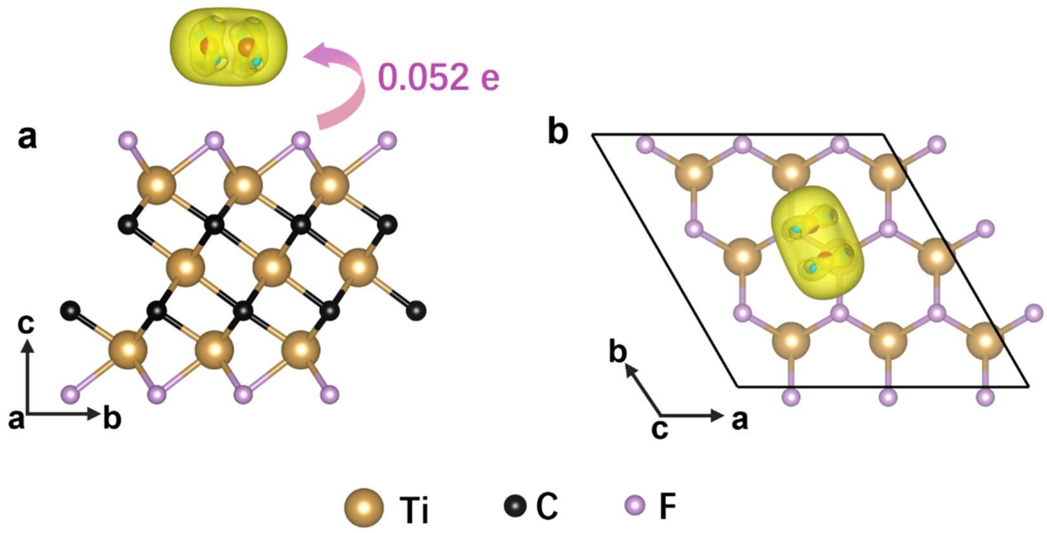

Fig. S15 The charge density difference plots for O_2 adsorbed on $Ti_3C_2F_2$: (a) side view, (b) top view. The isosurface level is set to be 0.0002 e/Å^3.

The E_{ad} of O_2 molecule adsorbed on $Ti_3C_2F_2$ is -0.030 eV, less negative than that on EB (-0.916 eV). This is in line with the transferred charges: the adsorbed O_2 obtains 0.421 electron from EB, much larger than that on $Ti_3C_2F_2$ (0.052 electron) (Fig. S14), further confirming the stronger binding ability between O_2 and EB. The results reveal that $Ti_3C_2F_2$ exhibits more inferior interaction with O_2 to EB, suggestive of similar behavior to $Ti_3C_2O_2$.

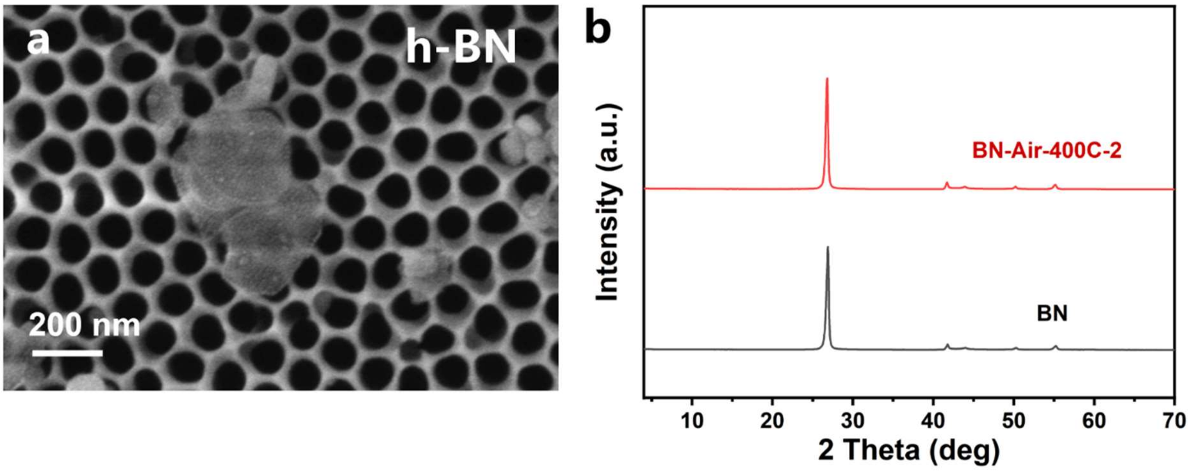

Fig. S16 (a) SEM image of the BN nanosheets. (b) XRD patterns of *h*-BN powder and *h*-BN powder treated at 400 ℃ for 2 hours in 90% RH Ar and synthetic air, which are confirm to PDF card 34-0421. Source data are provided as a Source Data file.

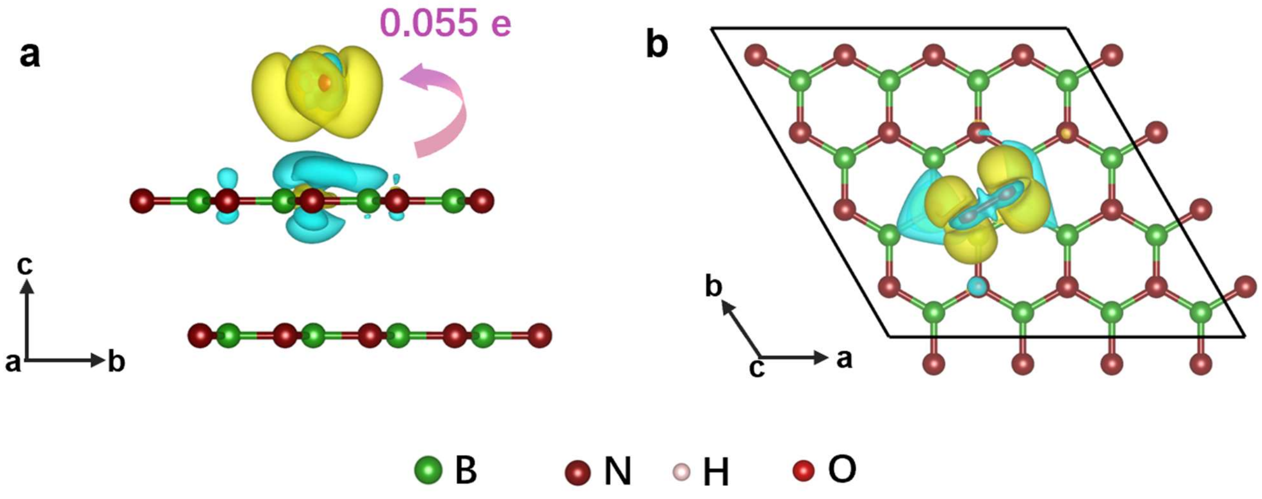

Fig. S17 The charge density difference plots for O_2 adsorbed on BN bilayer in systems: (a) side view, (b) top view. The isosurface level is set as 0.0002 e/Å^3.

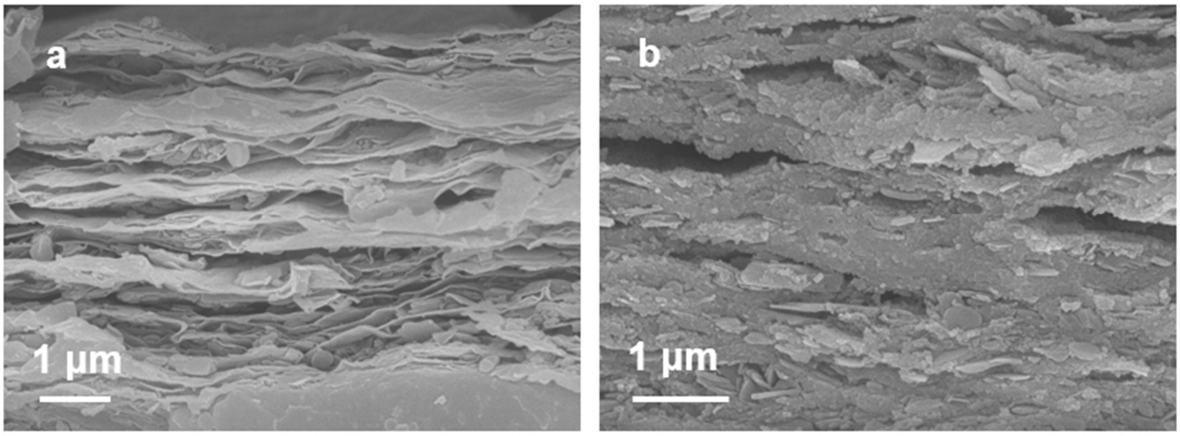

Fig. S18 SEM images of the cross-section of MBN before (a) and after (b) annealing at 400 °C for 2 hours in synthetic air.

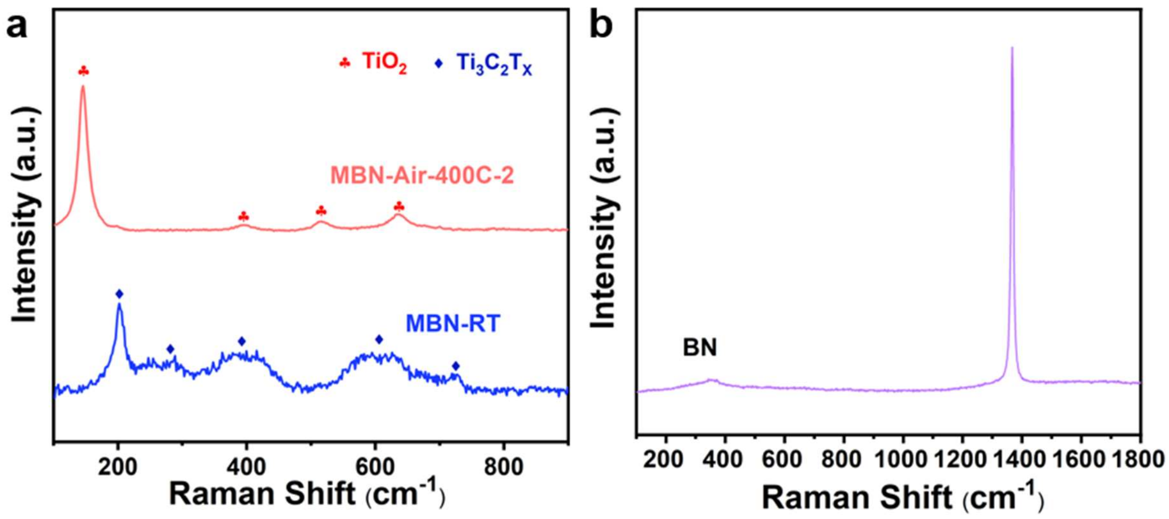

Fig. S19 Raman spectra of MBN-RT, MBN-Air-400C-2 (a) and *h*-BN (b). Source data are provided as a Source Data file.

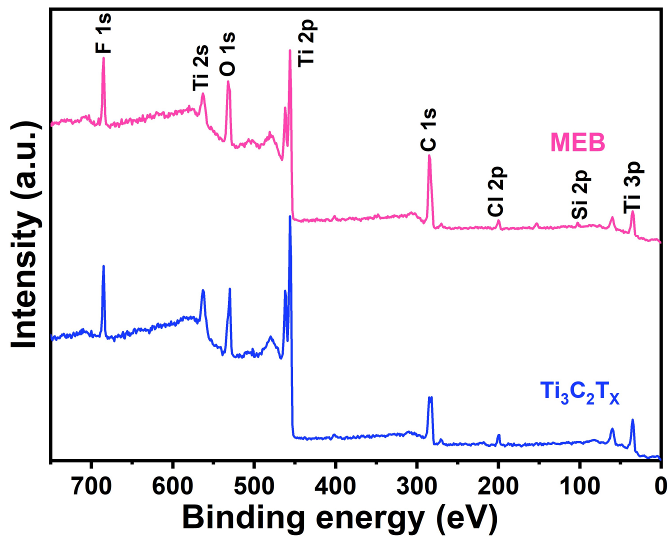

Fig. S20 XPS spectra of $Ti_3C_2T_x$ and MEB films. Source data are provided as a Source Data file.

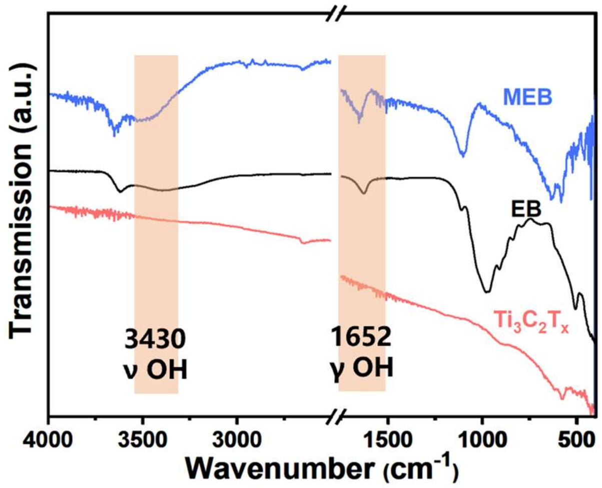

Fig. S21 Fourier transform infrared spectroscopy (FT-IR) of $Ti_3C_2T_x$, EB and MEB films. Source data are provided as a Source Data file.

Table S1. The permeabilities of water and oxygen for $Ti_3C_2T_x$ and MEB membranes

**Sample**	**Atmosphere**	**Permeability**
$Ti_3C_2T_x$	O_2	244713 $cm^3/(m^2 \cdot day)$
	H_2O	296 $g/(m^2 \cdot day)$
MEB	O_2	128856 $cm^3/(m^2 \cdot day)$
	H_2O	241 $g/(m^2 \cdot day)$

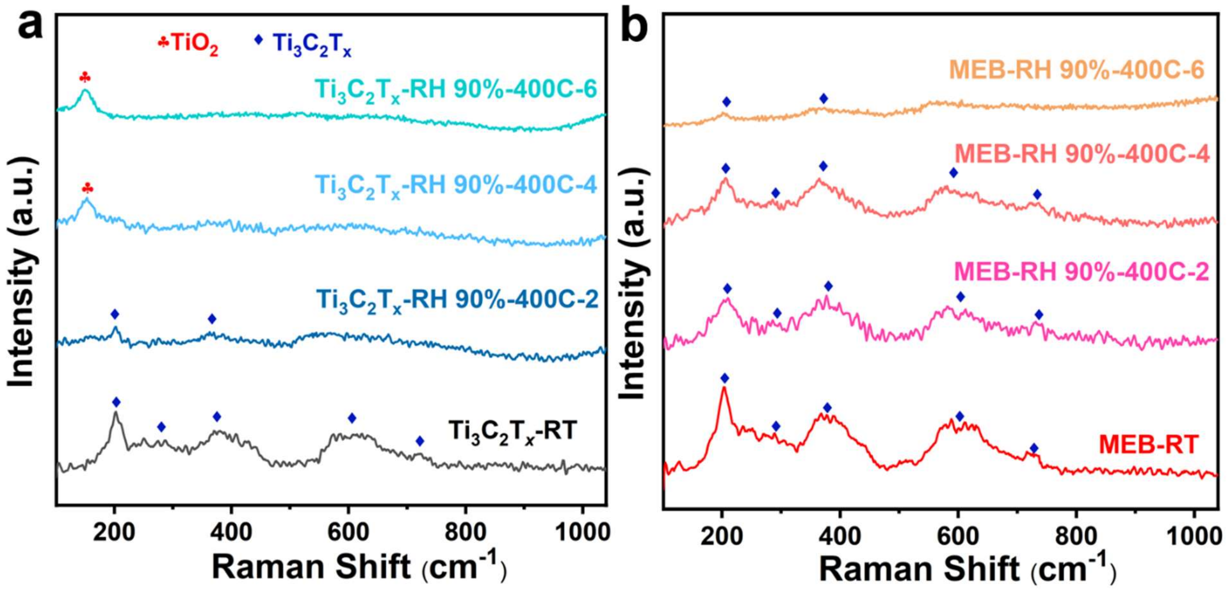

Fig. S22 (a) Raman spectra of $Ti_3C_2T_x$ films before and after treatment under RH 90% Ar at 400 ℃ for 2, 4, 6 hours. (b) Raman spectra of fresh MEB films before and after treatment under RH 90% Ar at 400 ℃ for 2, 4, 6 hours. Source data are provided as a Source Data file.

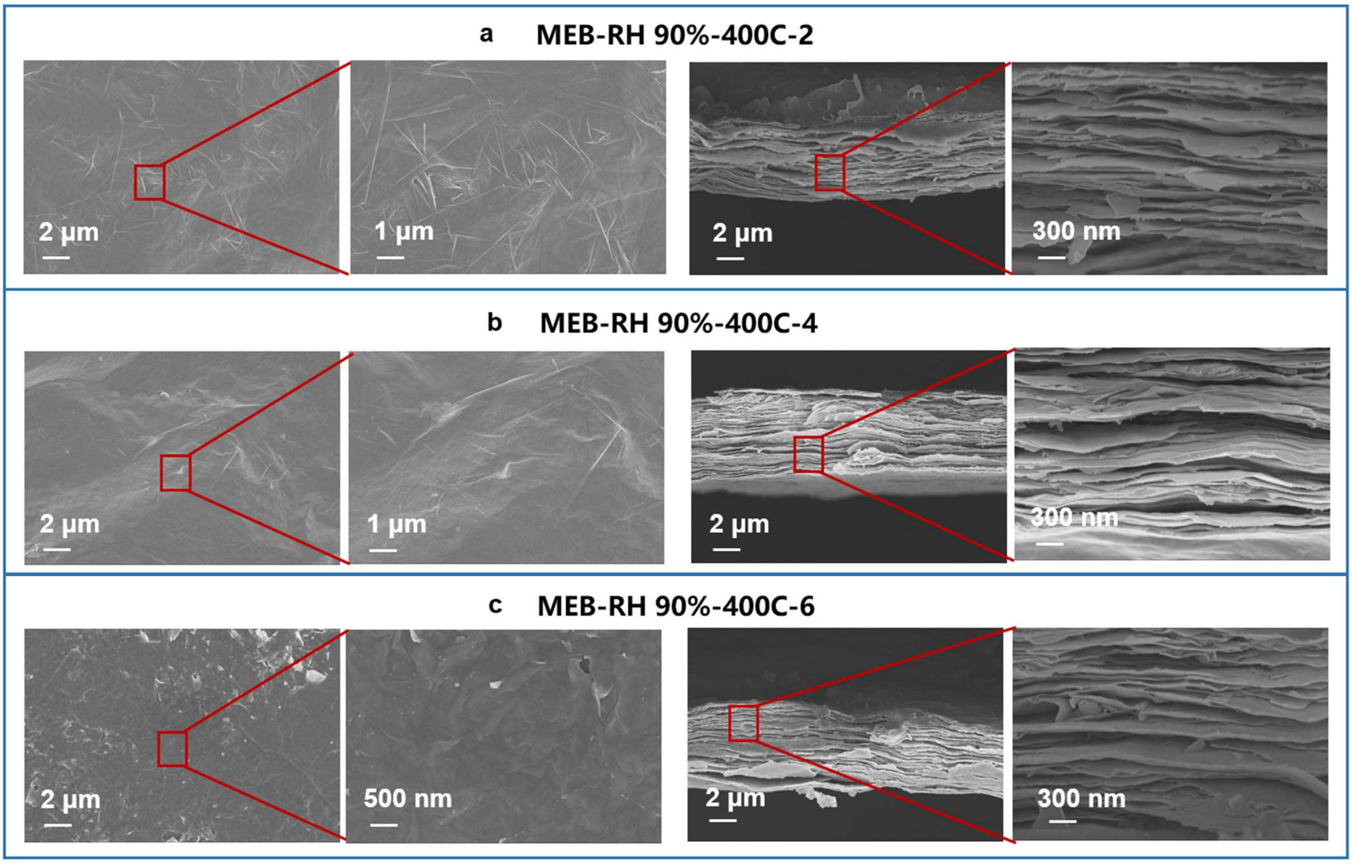

Fig. S23 SEM images of the surface and cross-section of different samples: (a) MEB-RH 90%-400C-2; (b) MEB- RH 90%-400C-4; (c) MEB- RH 90%-400C-6.

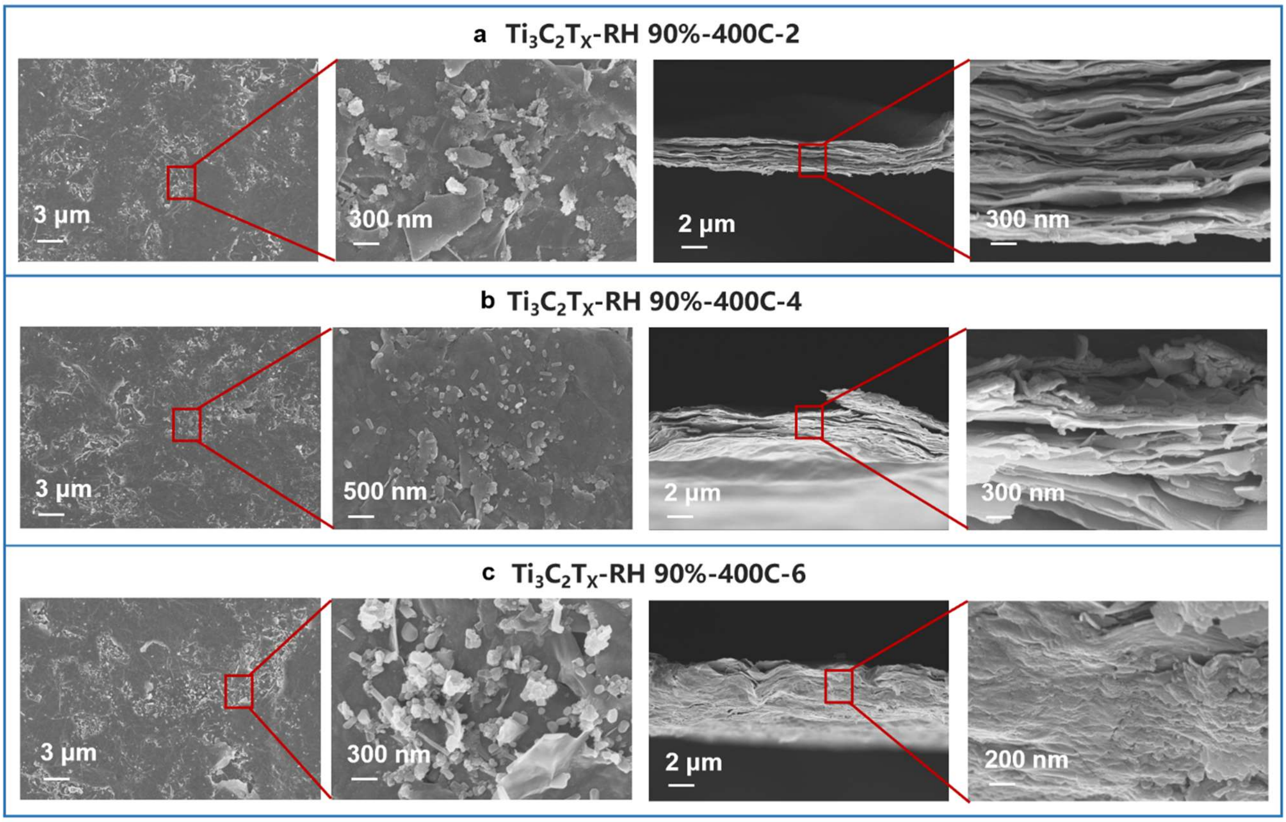

Fig. S24 SEM images of the surface and cross-section of different samples: (a) $Ti_3C_2T_x$-RH 90%-400C-2, (b) $Ti_3C_2T_x$-RH 90%-400C-4, (c) $Ti_3C_2T_x$-RH 90%-400C-6.

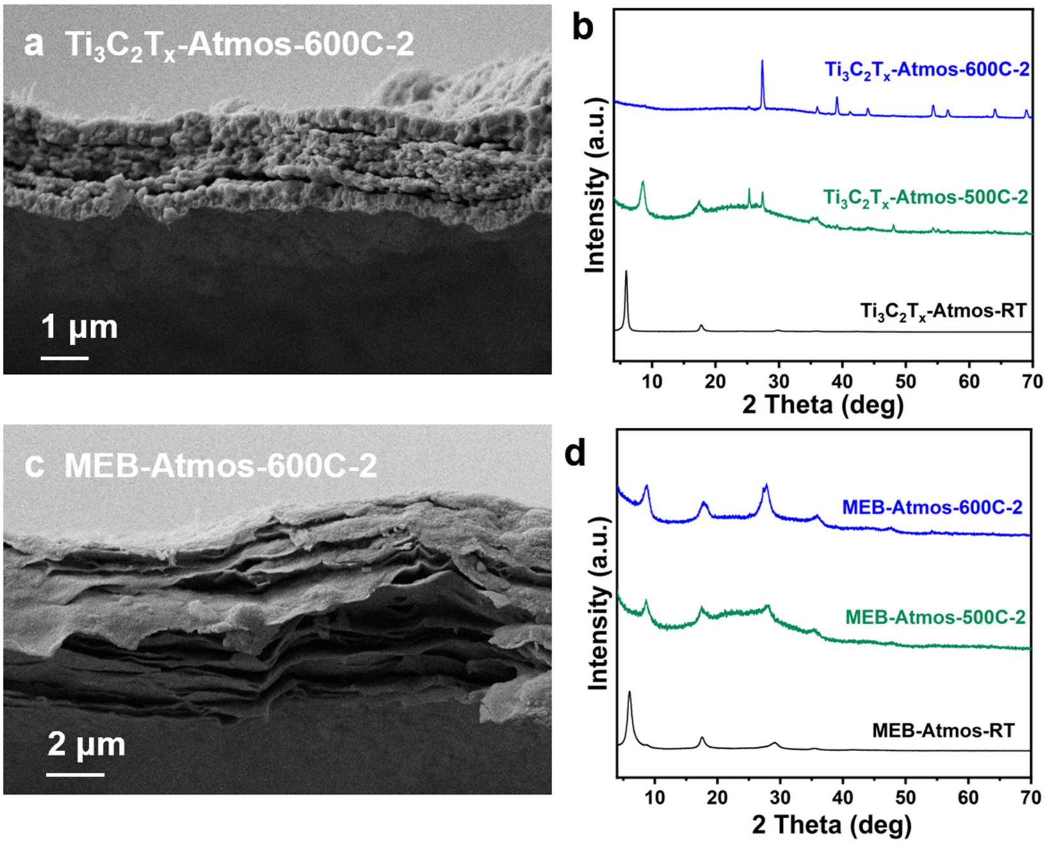

Fig. S25 (a) Cross-sectional SEM images of the $Ti_3C_2T_x$ film annealed in simulated atmospheric environment (Atoms) at 600 ℃ for 2 hours. (b) XRD patterns of $Ti_3C_2T_x$ after annealing under different conditions. (c) Cross-sectional SEM images of the MEB film annealed in simulated atmospheric environment (Atoms) at 600 ℃ for 2 hours. (d) XRD patterns of MEB after annealing under different conditions. Source data are provided as a Source Data file.

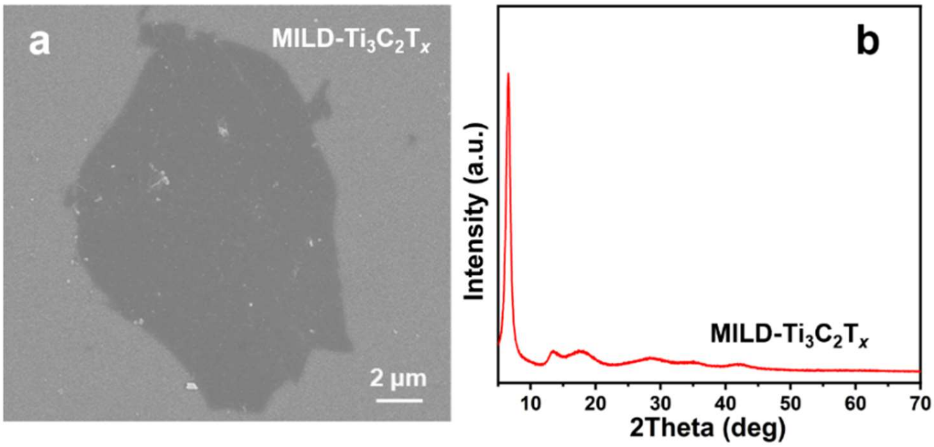

Fig. S26 (a) SEM image of MILD-$Ti_3C_2T_x$ flake; (b) XRD pattern of MILD-$Ti_3C_2T_x$. Source data are provided as a Source Data file.

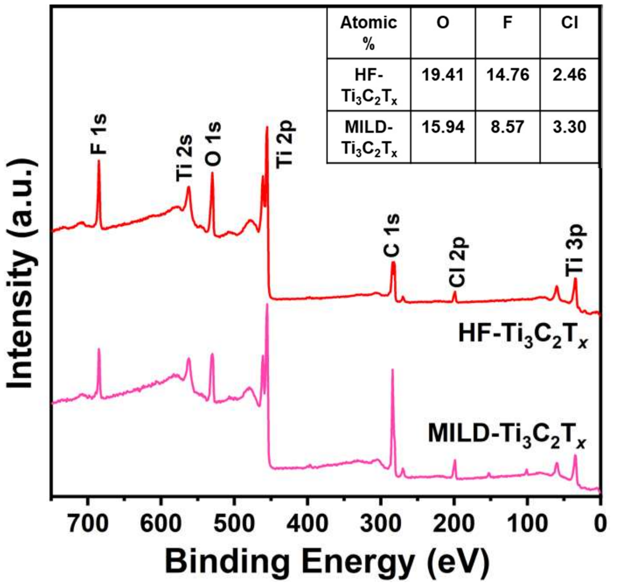

Fig. S27 XPS survey spectra of HF-$Ti_3C_2T_x$ and MILD-$Ti_3C_2T_x$ films with the ratio of O, F, Cl elements as a list (inserted). Source data are provided as a Source Data file.

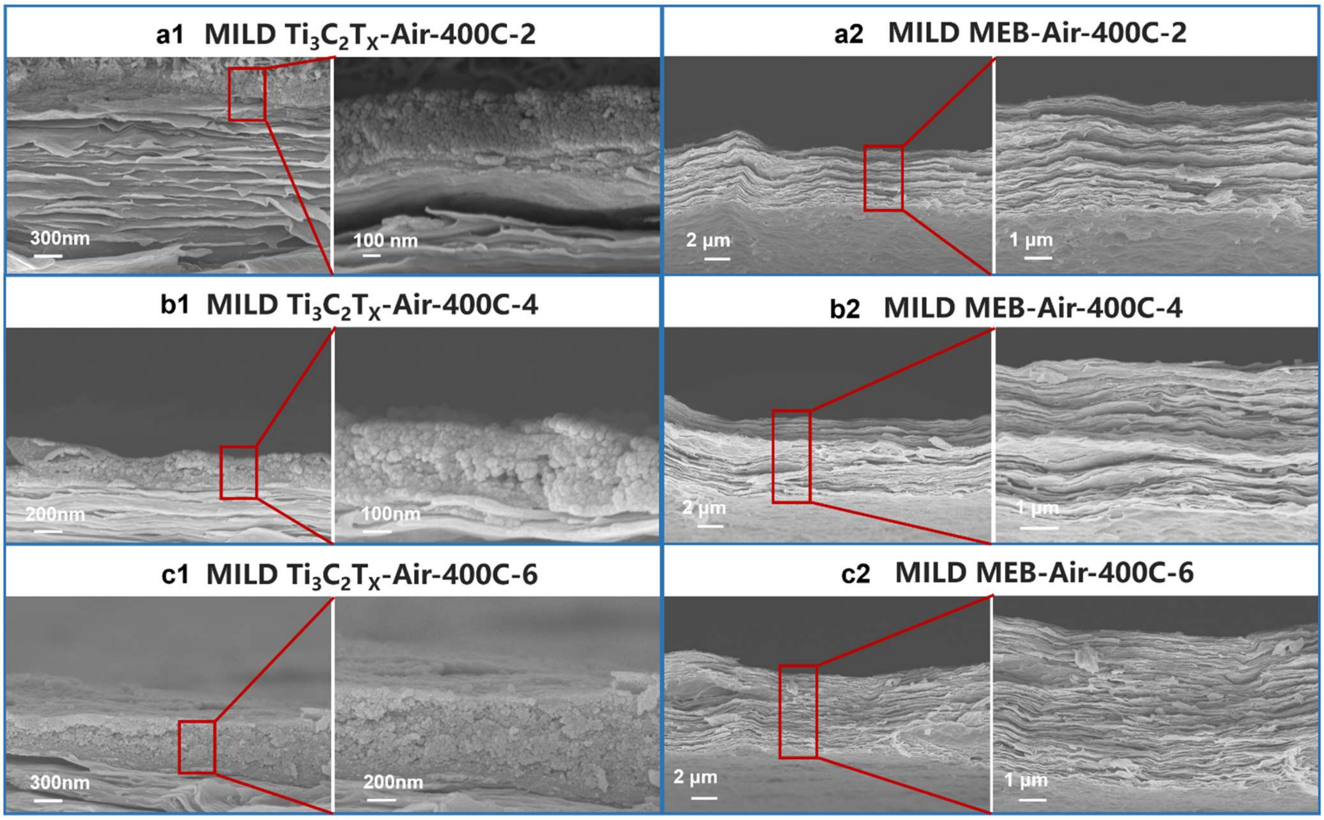

Fig. S28 SEM images of the cross-section of different samples: (a1) MILD $Ti_3C_2T_x$-Air-400C-2, (b1) MILD $Ti_3C_2T_x$-Air-400C-4, (c1) MILD $Ti_3C_2T_x$-Air-400C-6; (a2) MILD MEB-Air-400C-2, (b2) MILD MEB-Air-400C-4, (c2) MILD MEB-Air-400C-6.

The cross-sectional SEM images show a good retention of layered structure for MILD MEB-Air-400C-2, 4, 6. At the same time, TiO_2 particle is obvious in MILD $Ti_3C_2T_x$-Air-400C-2, 4, 6.

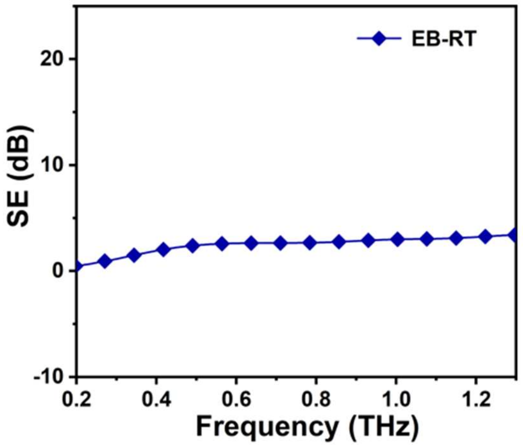

Fig. S29 THz SE in 0.2-1.3 THz of EB film. Source data are provided as a Source Data file.

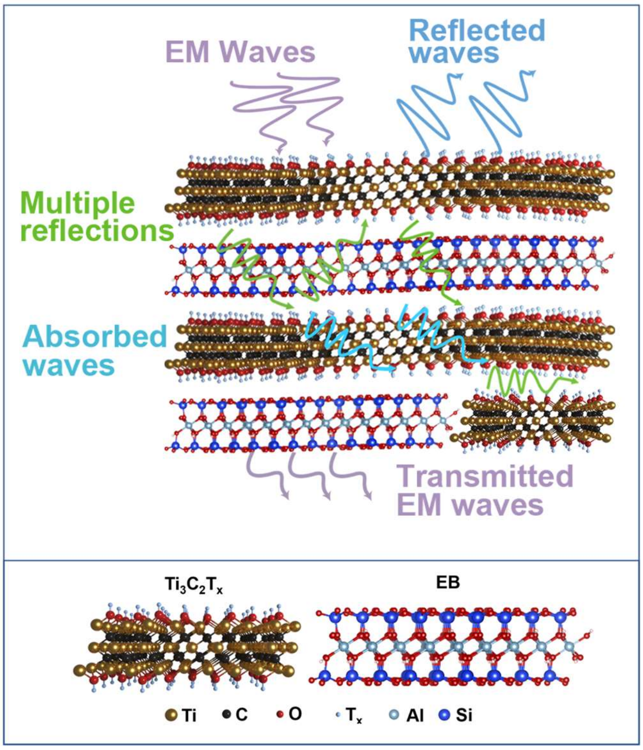

Fig. S30 Proposed EMI shielding mechanism of MEB, based on different shielding contributions (i.e. reflection, adsorption, multiple reflection). The EMI shielding originates from the high electrical conductivity of $Ti_3C_2T_x$ since EB is noneffective for THz shielding. Each time the intensity of an EM wave is decreased when transmitted through a MXene flake. As a result, the total EMI shielding effectiveness (EMI SE) is the sum of the effectiveness of all attenuating mechanisms, including absorption, reflection, and the multiple reflections[1-3].

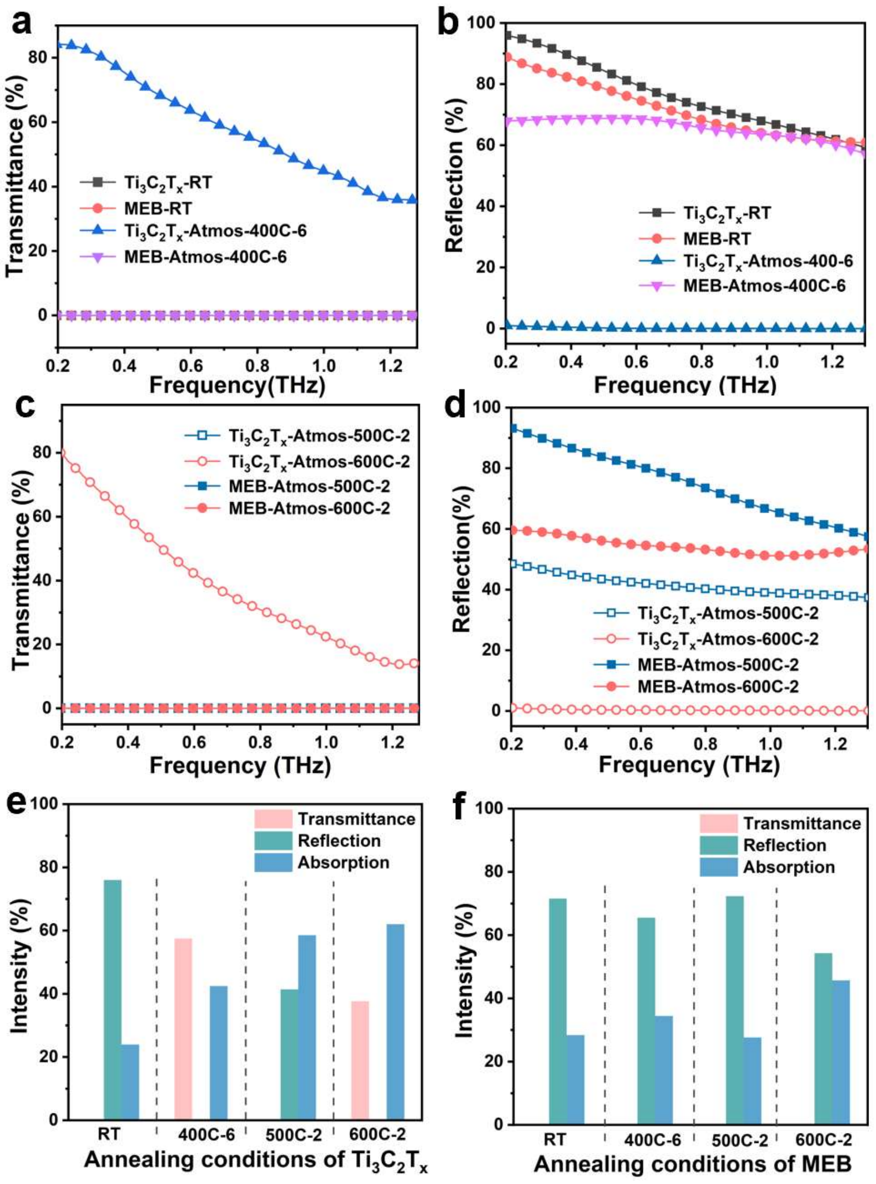

Fig. S31 High-temperature resistant THz shielding property. THz transmittance (a) and reflection (b) in 0.2-1.3 THz of $Ti_3C_2T_x$-RT, MEB-RT, $Ti_3C_2T_x$-Atmos-400C-6, MEB-Atmos-400C-6. THz transmittance (c) and reflection (d) in 0.2-1.3 THz of $Ti_3C_2T_x$-Atmos-500C, 600C-2 and MEB-Atmos-500C, 600C-2. The average THz transmittance, reflection, and absorption of $Ti_3C_2T_x$ (e) and MEB (f) before and after treatments. Source data are provided as a Source Data file.

It can be clearly observed that the transmitted THz signals of $Ti_3C_2T_x$-RT and MEB-RT are close to 0% (Fig. S31a), which is due to the excellent THz shielding performance of $Ti_3C_2T_x$ with the domination of THz reflection (Fig. S31b, e, f). After annealing, MEB-Atmos-400C-6 have a good shielding capacity retention, with a contribution of ~65% THz reflection and ~35% THz absorption (Fig. S31b, f). By contrast, $Ti_3C_2T_x$-Atmos-400C-6 performs high transmittance (~58%) and ultralow reflection (approach to zero) owing to its degradation. With the annealing temperature increasing, the transmissions of THz waves through MEB-

Atmos-500C-2h and MEB-Atmos-600C-2h remain at ~ 0%, with the decrease of reflection and increase of absorption. On the contrary, $Ti_3C_2T_x$-Atmos-600C-2h exhibits ~0 dB THz EMI shielding efficiency, because of the oxidation of $Ti_3C_2T_x$. All the results suggest that the introducing of EB can suppress the oxidation-induced deterioration of $Ti_3C_2T_x$, therefore making MEB being promising for THz shielding at high temperature in oxidizing environment for a long operation time.

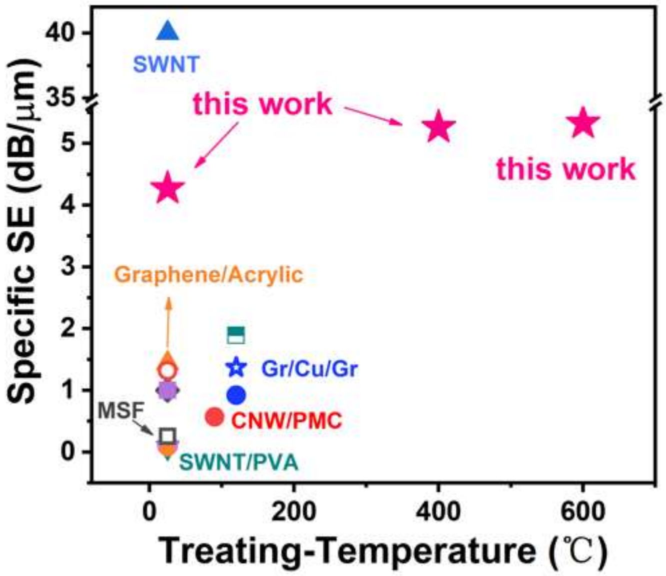

Fig. S32 Comparison of specific SE and treating temperature for various THz shielding materials. Each symbol indicates a set of material category as follows: CA-Graphene composites (deep grey open rhombus), CNW/PMC (red filled circle), SWNT (blue filled triangle), SWNT/PVA (green filled triangle), MWNT (purple filled square), Graphene/Acrylic (orange filled triangle), MXene/graphene/PDMS (purple circle), Cu/Graphene (green half-filled square), Gr/Cu/Gr (blue open star), Cu (blue filled circle), PUS-Ni/MXene (orange filled circle), MSF (deep grey open square), MXene waterborne painting (red circle), MEB in this work (pink star). A detailed description of each data point is presented in Table S2.

Table S2 Comparison of treating temperature for various THz shielding materials.

**Materials**	**Measured Band (THz)**	**Average SE (dB)**	**Thickness (μm)**	**Specific SE (dB/μm)**	**Treating Temperature (℃)**	**Ref.**
CA-Graphene composites	0.5-0.75	40	40	1.0	25	4
CNW/PMC	0.57-0.63	40	70	0.57	90	5
SWNT	0.2-2.5	38	0.95	40.0	25	6
SWNT/PVA	1.25-2.1	20	300	0.07	25	7
MWNT	0.4-2.2	25	25	1.0	25	8
Graphene/Acrylic	0.5-0.75	36	25	1.44	25	9
MXene/graphene/PDMS	0.2-2.0	45.3	400-500	0.11	25	10
Cu/Graphene	0.1-1.0	56.7	~ 30	1.89	120	11
Gr/Cu/Gr	0.1-1.0	41	~ 30	1.37	120	11
Cu	0.1-1.0	27.5	30	0.92	120	11
PUS-Ni/MXene	0.1-2.2	42.7	800	0.05	25	12
MSF	0.2-2.0	51	200	0.255	25	13
MXene waterborne painting	0.2-1.6	50.5	38.3	1.32	25	14
MEB	0.2-1.3	47	11	4.27	25	This work
MEB	0.2-1.3	50	9.5	5.26	400	This work
MEB	0.2-1.3	48	9.0	5.33	600	This work

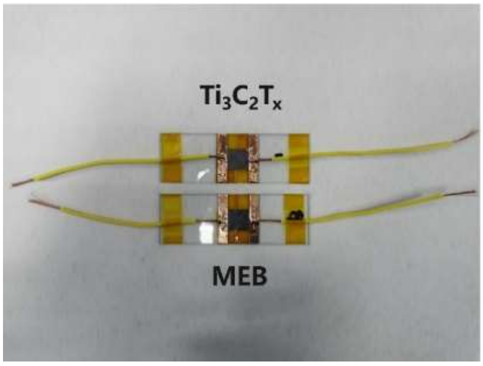

Fig. S33 The devices of Joule heater assembled by freestanding $Ti_3C_2T_x$ and MEB films.

Table S3 Comparison of the steady temperature and heating rate for various Joule heating materials.

**Materials**	**Method**	**Steady temperature (℃)**	**Heating rate (℃/s)**	**Voltage (V)**	**Application**	**Reference**
**Graphene fabric**	Spray-coating	162.6	8.4	10	Joule heater	15
**Graphene-EC on Ag NW-based film**	Spin-coating	99.8	3.4	12	Joule heater	16
**PEDOT/PSS film**	Deposition	100	1.6	12	Flexible transparent heater	17
**Graphene**	Electrochemical method	75	2.5	10	Ultrafast Electrothermal Heater	18
**MXene/aramid nonwoven fabric**	Spray coating	125	7.5	3	Electrothermal/photothermal conversion for wearable heater	19
		263	10	5		
**MXene patten**	Screen-printed	130.8	20	4	Joule heating	20
**MXene/cellulose fabric**	Dip coating	45	1	3	Healthcare and medical therapy	21
**MXene freestanding film**	Vacuum filtration	190	20	3	Joule heating	This work
**MEB freestanding film**	Vacuum filtration	198	20	3	Joule heating	This work

According to previous researches, $Ti_3C_2T_x$ MXene exhibits metallic conductivity (up to 2×10^4 S/cm) and high thermal conductivity [55.8 W/(m·K)] [22, 23], which meets the demand of Joule heating device on basis of Joule's law, $Q = I^2Rt = U^2t/R$ and $P = U^2/R$ where Q is the generated Joule heat, I is the DC current, U is the DC voltage, P is the output power of Joule heater, R is the resistance of film, t is the working time.

In our work, the $Ti_3C_2T_x$ and MEB freestanding films were used to make the Joule heater device, in which the resistances of $Ti_3C_2T_x$ and MEB are 2 Ω and 6 Ω, respectively (Fig. S33). The heating performance (including steady temperature and heating rate) of the films was conducted under a driving voltage of 3.0 V as shown in Fig. 5d. According to Joule's law, the heat is generated by the current flow through the films, which induced the temperature increase of the film. In our Joule heating devices, the output power of $Ti_3C_2T_x$ is 4.5 W and the output power of MEB is 1.5 W.

Furthermore, the comparison of the steady temperature and heating rate for various Joule heating materials was given in Table S3. The steady temperature of MEB is higher than MXene/aramid nonwoven fabric and MXene/cellulose fabric under the driving voltage of 3 V. Besides, the heating rate of MEB in our work is 20 °C s^{-1}, much higher than graphene/MXene fabrics and PEDOT/PSS film shown in Table S3.

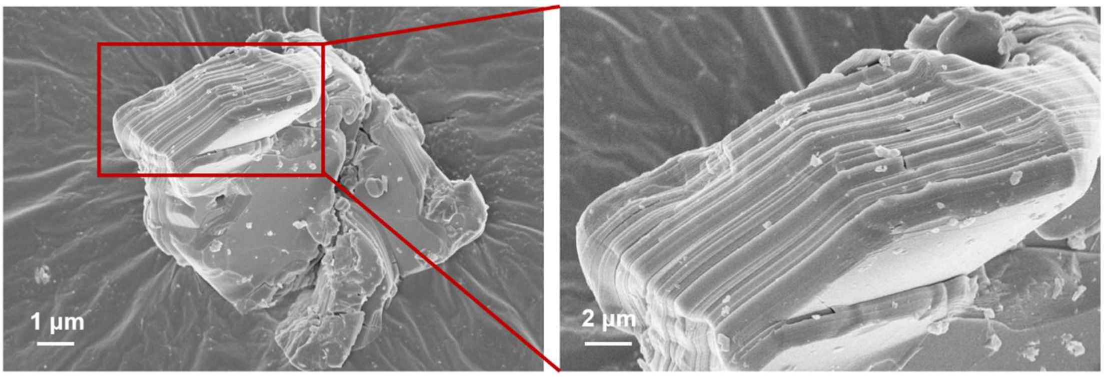

Fig. S34 SEM image of a square Ti_3AlC_2 particle. The Ti_3AlC_2 particle (≈500 μm^3) is predominately made up of square, platelet-like particles, which is consistent with the literatures report[25, 26]

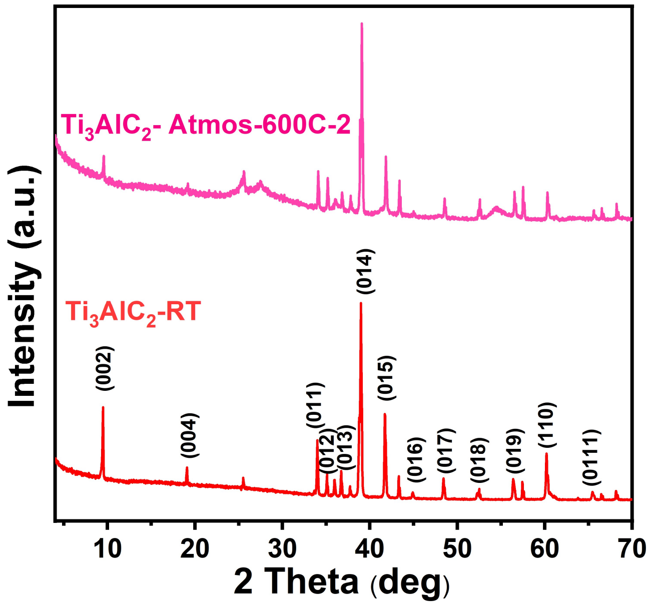

Fig. S35 XRD patterns of Ti_3AlC_2-RT and Ti_3AlC_2-Atmos-600C-2 (annealing under 60% RH air at 600°C for 2h). The XRD patterns of Ti_3AlC_2-RT and Ti_3AlC_2-Atmos-600C-2 show the similar peaks with high intensity of (002) (011) (012) (014) (015) and (110) [27-29], which confirms the stability of Ti_3AlC_2 at 600°C. Source data are provided as a Source Data file.

Supplementary References

1. Shahzad, F. *et al.* Electromagnetic interference shielding with 2D transition metal carbides (MXene). *Science* **353**, 1137-1140 (2016).
2. Chen, Z., Xu, C., Ma, C., Ren, W. & Cheng, H. M. Lightweight and flexible graphene foam composites for high-performance electromagnetic interference shielding. *Adv. Mater.* **25**, 1296-1300 (2013).
3. Pavlou, C. *et al.* Effective EMI shielding behaviour of thin graphene/PMMA nanolaminates in the THz range. *Nat. Commun.* **12**, 4655 (2021).
4. Cataldi, P. et al. Cellulosic graphene biocomposites for versatile high-performance flexible electronic applications. *Adv. Electron. Mater.* **2**, 1600245 (2016).
5. Das, A., et al. Quasi-optical terahertz polarizers enabled by inkjet printing of carbon nanocomposites. *Appl. Phys. Lett.* **101,** 243108 (2012).
6. Hong, J. T. al et. Terahertz wave applications of single-walled carbon nanotube films with high shielding effectiveness. *Appl. Phys. Express* **5**, 015102 (2012).
7. Polley, D., Barman, A. & Mitra, R. K. EMI shielding and conductivity of carbon nanotube-polymer composites at terahertz frequency. *Opt. Lett.* **39**, 1541-1544 (2014).
8. Polley, D., Neeraj, K., Barman, A. & Mitra, R. K. Diameter-dependent shielding effectiveness and terahertz conductivity of multiwalled carbon nanotubes. *J. Opt. Soc. Am. B* **33**, 2430-2436 (2016).
9. Mates, J. E., et al. Durable and flexible graphene composites based on artists' paint for conductive paper applications. *Carbon* **87**, 163-174 (2015).
10. Nguyen, V.-T., Min, B. K., Yi, Y., Kim, S. J. & Choi, C.-G. MXene($Ti_3C_2T_X$)/graphene/PDMS composites for multifunctional broadband electromagnetic interference shielding skins. *Chem. Eng. J.* **393**, 124608 (2020).
11. Hou, S. al et. Excellent Terahertz shielding performance of ultrathin flexible Cu/graphene nanolayered composites with high stability. *J. Mater. Sci. Technol.* **52**, 136-144 (2020).
12. Bai, Y., Qin, F. & Lu, Y., Flexible and lightweight Ni/MXene decorated polyurethane sponge composite with sensitive strain sensing performance for ultrahigh terahertz absorption. *Adv. Opt. Mater.*, 2101868 (2021).
13. Shui, W. al et. $Ti_3C_2T_x$ MXene sponge composite as broadband terahertz absorber. *Adv. Opt. Mater.* **8**, 2001120 (2020).
14. Wan, H., Liu, N., Tang, J., Wen, Q. & Xiao, X. Substrate-independent $Ti_3C_2T_x$ MXene waterborne paint for terahertz absorption and shielding. *ACS Nano* **15**, 13646-13652 (2020).
15. Tian, M. *et al.* Enhanced electrothermal efficiency of flexible graphene fabric Joule heaters with the aid of graphene oxide. *Mater. Lett.* **234**, 101-104 (2019).
16. Cao, M., Wang, M., Li, L., Qiu, H. & Yang, Z. Effect of graphene-EC on Ag NW-based transparent film heaters: optimizing the stability and heat dispersion of films. *ACS Appl. Mater. Interfaces* **10**, 1077-1083 (2018).
17. Gueye, M. N., Carella, A., Demadrille, R. & Simonato, J. P. All-polymeric flexible transparent heaters. *ACS Appl. Mater. Interfaces* **9**, 27250-27256 (2017).
18. Tian, S. *et al.* Electrochemical fabrication of high quality graphene in mixed electrolyte for ultrafast electrothermal heater. *Chem. Mater.* **29**, 6214−6219 (2017).
19. Wang, X. *et al.* A lightweight MXene-coated nonwoven fabric with excellent flame retardancy, EMI shielding, and electrothermal/photothermal conversion for wearable heater. *Chem. Eng. J.* **430**, (2022).

20. Wu, H. *et al.* Aqueous MXene/Xanthan Gum hybrid inks for screen-printing electromagnetic shielding, Joule heater, and piezoresistive sensor. *Small* e2107087 (2022).

21. Zhao, X. *et al.* Smart $Ti_3C_2T_x$ MXene fabric with fast humidity response and Joule heating for healthcare and medical therapy applications. *ACS Nano* **14**, 8793-8805 (2020).

22. Guofa Cai, Jing-Hao Ciou, Yizhi Liu, Yi Jiang & Pooi See Lee. Leaf-inspired multiresponsive MXene-based actuator for programmable smart devices. *Sci. Adv.* **5**, eaaw7956 (2019).

23. Mathis, T. S. et al. Modified MAX phase synthesis for environmentally stable and highly conductive Ti_3C_2 MXene. *ACS Nano* 15, 6420-6429 (2021).

24. Zhao, X. et al. Smart $Ti_3C_2T_x$ MXene fabric with fast humidity response and Joule heating for healthcare and medical therapy applications. *ACS Nano* **14**, 8793-8805 (2020).

25. Naguib, M. *et al.* Two-dimensional nanocrystals produced by exfoliation of Ti_3AlC_2. *Adv. Mater.* **23**, 4248-4253 (2011).

26. Mathis, T. S. *et al.* Modified MAX phase synthesis for environmentally stable and highly conductive Ti_3C_2 MXene. *ACS Nano* **15**, 6420-6429 (2021).

27. Tasleem, S., Tahir, M. & Zakaria, Z. Y. Fabricating structured 2D Ti_3AlC_2 MAX dispersed TiO_2 heterostructure with Ni_2P as a cocatalyst for efficient photocatalytic H2 production. *J. Alloys Compd.* **842**, (2020).

28. Khan, A. A. & Tahir, M. Well-designed 2D/2D $Ti_3C_2T_{A/R}$ MXene coupled g-C_3N_4 heterojunction with in-situ growth of anatase/rutile TiO_2 nucleates to boost photocatalytic dry-reforming of methane (DRM) for syngas production under visible light. *Appl. Catal. B Environ.* **285**, 119777 (2021).

29. Tahir, M. & Tahir, B. In-situ growth of TiO_2 imbedded $Ti_3C_2T_A$ nanosheets to construct PCN/$Ti_3C_2T_A$ MXenes 2D/3D heterojunction for efficient solar driven photocatalytic CO_2 reduction towards CO and CH_4 production. *J. Colloid Interface Sci.* **591**, 20-37 (2021).
