# Supplementary material for: High-temperature stability in air of Ti3C2Tx MXene-based composite with extracted bentonite
Source: Nat Commun. 2022 Sep 22;13:5551. doi: 10.1038/s41467-022-33280-2 (PMC9499972; doi:10.1038/s41467-022-33280-2)
Supplement: Supplementary file 3 — Reporting Summary [file 41467_2022_33280_MOESM3_ESM.pdf]

nature portfolio

Corresponding author(s): Xu Xiao

Last updated by author(s): Sep 7, 2022

Reporting Summary

Nature Portfolio wishes to improve the reproducibility of the work that we publish. This form provides structure for consistency and transparency in reporting. For further information on Nature Portfolio policies, see our Editorial Policies and the Editorial Policy Checklist.

Statistics

For all statistical analyses, confirm that the following items are present in the figure legend, table legend, main text, or Methods section.

n/a	Confirmed	
☐	☒	The exact sample size (*n*) for each experimental group/condition, given as a discrete number and unit of measurement
☐	☒	A statement on whether measurements were taken from distinct samples or whether the same sample was measured repeatedly
☐	☒	The statistical test(s) used AND whether they are one- or two-sided<br>*Only common tests should be described solely by name; describe more complex techniques in the Methods section.*
☐	☒	A description of all covariates tested
☐	☒	A description of any assumptions or corrections, such as tests of normality and adjustment for multiple comparisons
☐	☒	A full description of the statistical parameters including central tendency (e.g. means) or other basic estimates (e.g. regression coefficient) AND variation (e.g. standard deviation) or associated estimates of uncertainty (e.g. confidence intervals)
☐	☒	For null hypothesis testing, the test statistic (e.g. *F*, *t*, *r*) with confidence intervals, effect sizes, degrees of freedom and *P* value noted<br>*Give P values as exact values whenever suitable.*
☐	☒	For Bayesian analysis, information on the choice of priors and Markov chain Monte Carlo settings
☐	☒	For hierarchical and complex designs, identification of the appropriate level for tests and full reporting of outcomes
☐	☒	Estimates of effect sizes (e.g. Cohen's *d*, Pearson's *r*), indicating how they were calculated

Our web collection on statistics for biologists contains articles on many of the points above.

Software and code

Policy information about availability of computer code

Data collection	*Provide a description of all commercial, open source and custom code used to collect the data in this study, specifying the version used OR state that no software was used.*
Data analysis	*Provide a description of all commercial, open source and custom code used to analyse the data in this study, specifying the version used OR state that no software was used.*

For manuscripts utilizing custom algorithms or software that are central to the research but not yet described in published literature, software must be made available to editors and reviewers. We strongly encourage code deposition in a community repository (e.g. GitHub). See the Nature Portfolio guidelines for submitting code & software for further information.

Data

Policy information about availability of data

All manuscripts must include a data availability statement. This statement should provide the following information, where applicable:

- Accession codes, unique identifiers, or web links for publicly available datasets
- A description of any restrictions on data availability
- For clinical datasets or third party data, please ensure that the statement adheres to our policy

The datasets generated during and/or analysed during the current study are available from the corresponding author on reasonable request.

Human research participants

Policy information about studies involving human research participants and Sex and Gender in Research.

Reporting on sex and gender	*Use the terms sex (biological attribute) and gender (shaped by social and cultural circumstances) carefully in order to avoid confusing both terms. Indicate if findings apply to only one sex or gender; describe whether sex and gender were considered in study design whether sex and/or gender was determined based on self-reporting or assigned and methods used. Provide in the source data disaggregated sex and gender data where this information has been collected, and consent has been obtained for sharing of individual-level data; provide overall numbers in this Reporting Summary. Please state if this information has not been collected. Report sex- and gender-based analyses where performed, justify reasons for lack of sex- and gender-based analysis.*
Population characteristics	*Describe the covariate-relevant population characteristics of the human research participants (e.g. age, genotypic information, past and current diagnosis and treatment categories). If you filled out the behavioural & social sciences study design questions and have nothing to add here, write "See above."*
Recruitment	*Describe how participants were recruited. Outline any potential self-selection bias or other biases that may be present and how these are likely to impact results.*
Ethics oversight	*Identify the organization(s) that approved the study protocol.*

Note that full information on the approval of the study protocol must also be provided in the manuscript.

Field-specific reporting

Please select the one below that is the best fit for your research. If you are not sure, read the appropriate sections before making your selection.

☐ Life sciences ☐ Behavioural & social sciences ☒ Ecological, evolutionary & environmental sciences

For a reference copy of the document with all sections, see nature.com/documents/nr-reporting-summary-flat.pdf

Ecological, evolutionary & environmental sciences study design

All studies must disclose on these points even when the disclosure is negative.

Study description	Although Ti3C2Tx MXene is promising for catalysis, energy storage, electromagnetic interference shielding due to its metallic conductivity and high processability, it is not able to work under harsh environment due to the quick oxidation induced by oxygen at high temperature. Here, we report an air-stable Ti3C2Tx based composite (MEB) by compositing Ti3C2Tx with extracted bentonite (EB) nanosheets. Interestingly, oxygen molecules are preferentially adsorbed on EB. The saturated adsorption of oxygen on EB further inhibits more oxygen molecules to be absorbed on the surface of Ti3C2Tx due to the weakened p-d orbital hybridization between adsorbed O2 and Ti3C2Tx, which is induced by the Ti3C2Tx/EB interface coupling. As a result, MEB is capable of tolerating the high temperature annealing (above 400 °C for several hours) no matter in air or humid environment, indicating highly improved antioxidation in harsh condition. The above finding is independent on the termination ratio of Ti3C2Tx obtained through different synthesis routes. Utilized as terahertz shielding materials, MEB is able to work after high-temperature treatment even up to 600 °C, while pristine Ti3C2Tx is completely oxidized with no terahertz shielding ability. Joule heating and thermal cycling performance of MEB are also demonstrated.
Research sample	Ti3C2Tx based composite (MEB) by compositing Ti3C2Tx with extracted bentonite (EB) nanosheets.
Sampling strategy	XRD, SEM, STEM, Raman, XPS, TEM, IR are used to characterize the materials. The measurements are useful to understand the structure and the component of the materials.
Data collection	The datas are collected by all the authors though the characterization of the instruments.
Timing and spatial scale	0.2-1.3THz of the EMI shielding performance; 100-1000 cm-1 of Raman; 100-1000 degree of TG;℃℃
Data exclusions	No data exclusions
Reproducibility	The experiments are conducted more than two times and the reproducibility is good.
Randomization	The materials were allocated into groups by the components.
Blinding	Not applicable

Did the study involve field work? ☐ Yes ☒ No

Reporting for specific materials, systems and methods

We require information from authors about some types of materials, experimental systems and methods used in many studies. Here, indicate whether each material, system or method listed is relevant to your study. If you are not sure if a list item applies to your research, read the appropriate section before selecting a response.

Materials & experimental systems

n/a	Involved in the study
☒	☐ Antibodies
☒	☐ Eukaryotic cell lines
☒	☐ Palaeontology and archaeology
☒	☐ Animals and other organisms
☒	☐ Clinical data
☒	☐ Dual use research of concern

Methods

n/a	Involved in the study
☒	☐ ChIP-seq
☒	☐ Flow cytometry
☒	☐ MRI-based neuroimaging
